# Occlusal Relationships and Dental Changes in Mixed Dentition Patients Treated with Clear Aligners: A 2-Year Follow Up

**DOI:** 10.3390/children13020298

**Published:** 2026-02-21

**Authors:** Francesca Gazzani, Chiara Pavoni, Francesca Chiara De Razza, Letizia Lugli, Saveria Loberto, Alessio Lachi, Paola Cozza, Roberta Lione

**Affiliations:** Department of Health Science, UniCamillus-Saint Camillus International Medical University, 00131 Rome, Italy; francesca.gazzani@unicamillus.org (F.G.); chiara.pavoni@unicamillus.org (C.P.); francesca.derazza@unicamillus.org (F.C.D.R.); saveria.loberto@unicamillus.org (S.L.); alessio.lachi@unicamillus.org (A.L.); paola.cozza@unicamillus.org (P.C.); roberta.lione@unicamillus.org (R.L.)

**Keywords:** clear aligners, mixed dentition, interceptive orthodontics, arch expansion, long-term stability

## Abstract

**Highlights:**

**What are the main findings?**

- Clear aligner treatment in early mixed dentition resulted in statistically significant increases in transverse arch dimension, controlled molar derotation, torque modification, and overjet reduction.
- The dentoalveolar and occlusal changes achieved at the end of active treatment remained stable over a 2-year follow-up, with only minimal and non-significant relapse.

**What are the implications of the main findings?**

- Clear aligners represent an effective interceptive orthodontic modality for managing transverse discrepancies and anterior irregularity during growth.
- The demonstrated long-term stability, even in the absence of retention, supports the role of aligner-based protocols in early dentoalveolar correction and growth-guided orthodontic therapy.

**Abstract:**

Background: This study assessed the long-term stability of dental arch changes achieved through clear aligner treatment in growing patients during the early mixed dentition stage. Methods: This retrospective study included 20 patients (mean age 8.3 ± 0.4 years) treated with clear aligners according to a standardized sequential expansion protocol. No additional auxiliaries, interproximal reductions, or retentions were used. Dental casts were collected at baseline (T0), end of treatment (T1), and two years post-treatment without retention (T2). Linear and angular measurements (arch width, molar and incisor torque, Henry’s angle, overjet, overbite, and Little’s index) were assessed on digital models. Friedman ANOVA and Wilcoxon signed-rank tests were applied (α = 0.05). Results: At T1-T0, significant transversal expansion was achieved in both arches (U6–6 mesial +2.1 mm; L6–6 mesial +2.4 mm; *p* < 0.05), with favorable torque changes and a reduction in overjet (−1.5 mm). From T1 to T2, only minimal, non-significant relapse was detected, except for a slight reduction in lower left molar torque (−1.1°). The T2-T0 comparison confirmed stable improvements in mesial intermolar widths (upper +2.0 mm; lower +1.6 mm), molar derotations, and overjets (−1.9 mm), with no significant loss of expansion or sagittal correction. Conclusions: Clear aligners in early mixed dentition achieved significant and stable dental arch modifications over a 2-year follow-up without the use of retention appliances. This therapeutic approach may represent a reliable interceptive option in growing patients.

## 1. Introduction

Early orthodontic treatment, also known as phase I, aims to prevent the development of malocclusion and correct existing or developing skeletal, dentoalveolar, and muscular imbalances [1]. The interceptive approach improves the orofacial environment, allowing a more stable outcome compared to starting treatment at a later stage [2,3,4]. Moreover, malocclusions such as anterior or posterior crossbites, dento-skeletal Class III, Class II malocclusions with increased overjet, open-bite, and dento-basal discrepancies associated with tooth eruption disorders should be addressed promptly to promote proper occlusion development [1,5]. A number of authors disagree regarding the effectiveness of an early approach due to relevant costs, time and effort involved, and whether it offers better results compared to a one-stage treatment approach [4]. On the other hand, several authors have highlighted the positive effects of interceptive treatment in terms of crowding management, transversal arch expansion, and sagittal discrepancy correction [6,7,8,9,10,11]. More specifically, few studies and systematic reviews have focused on the long-term effects of upper and lower arch expansion achieved using rapid maxillary expansion and slow expansion devices [6,7,8,9,10,11,12,13]. The main findings indicate that changes in maxillary width tend to remain quite stable, with only slight relapse observed following rapid maxillary expansion treatment [12]. According to some longitudinal studies, transversal arch dimensions show a more pronounced relapse, retaining approximately 40% of initial molar expansion [6,14,15,16]. Regarding slow expansion protocols, the existing literature illustrates variable results, although most studies report a good long-term stability [17,18,19]. In recent years, interest in the use of clear aligners for growing patients, including mixed and primary dentition treatments, has progressively increased [20]. Recent studies revealed predictable results in terms of arch expansion and alignment with clear aligner treatment in early mixed dentition [21,22,23,24,25,26,27]. Levrini et al. [24] underscored significant improvement in transversal dimensions in 20 patients treated with clear aligners by means of 3D superimposition analysis, concluding that the forces generated by clear aligners are comparable to those obtained by slow maxillary expanders. Furthermore, Cretella Lombardo et al. [28] reported favorable results when comparing the expansion treatment performed with clear aligners in mixed dentition with an RME effect. However, while numerous studies [21,22,23,24,25,26,27,28] focused on the short-term effects of clear aligner treatment in mixed dentition patients, no data is available as to long-term stability.

Therefore, the aim of the present study was to evaluate the long-term stability of dental arch changes achieved through clear aligner treatment in growing patients during the early mixed dentition stage.

## 2. Materials and Methods

### 2.1. Subjects

This study project was authorized by the Ethical Committee of Saint Camillus International University of Health Sciences (Protocol number: E00178-2025) and a consent form was signed by all parents. The initial sample consisted of 55 subjects treated in a private practice context from January 2021 to December 2022.

### 2.2. Inclusion Criteria

To be included in the study, patients had to present with the following inclusion criteria: (1) early mixed dentition with fully erupted first permanent molars; (2) anterior dental crowding; (3) a transverse discrepancy between the maxillary and mandibular arches not exceeding 6 mm, with or without posterior crossbite; (4) adequate compliance with aligner treatment. The initial anterior crowding was measured using a modified version of Little’s irregularity index (LII) [29] applied to both the mandibular and maxillary arches during the mixed dentition phase. The index was calculated only with the four permanent incisors fully erupted and stable. For each dental arch, the total irregularity score was calculated as the sum of the horizontal linear distances measured at the anatomical contact points between adjacent anterior teeth using a digital caliper with a precision of 0.01 mm. This adaptation of the LII to the maxillary arch and to the mixed dentition period allows for a consistent and objective assessment of anterior alignment in growing patients. Although the LII was not originally designed for the upper arch or for mixed dentition, its use in this context has been previously described in the literature, particularly when the evaluation focuses on the permanent incisors and early interceptive orthodontic treatment planning [30]. Based on the severity of anterior crowding, the Little’s irregularity index (LII) was categorized into five levels: values <0.5 mm indicated ideal alignment; values between 0.5 and <3.5 mm indicated minimal irregularity; values between 3.5 and <6.5 mm indicated moderate irregularity; values between 6.5 and <9.5 mm indicated severe irregularity; and values ≥9.5 mm indicated very severe irregularity [29]. The selected study group showed a mandibular anterior crowding equivalent to a severe irregularity 6.5 ≤ LII < 9.5 mm.

### 2.3. Exclusion Criteria

Exclusion criteria included: (1) early loss of primary canines or molars; (2) advanced dental caries; (3) previous orthodontic interventions or adjunctive appliances; (4) dental agenesis or supernumerary teeth; (5) incomplete documentation; (6) poor compliance with aligner treatment. After application of the exclusion criteria, the final study sample consisted of 20 patients (12 females and 8 males), with a mean age of 8.3 ± 0.4 years.

### 2.4. Treatment Protocol

All patients underwent treatment with clear aligners using the Invisalign First System^®^ (Align Technology, Santa Clara, CA, USA). The treatment planning software automatically generated optimized expansion support and retention attachments positioned on the buccal surfaces of the teeth. No additional attachments beyond those automatically prescribed were applied to enhance the expansion protocol. Throughout treatment, no auxiliary appliances, interproximal enamel reductions (IPRs), or dental extractions were performed. A standardized sequential expansion protocol was planned for each patient. The protocol included the initial movement of the first permanent molars followed by the simultaneous expansion of all deciduous molars and canines (“molars move first” expansion pattern). During each expansion phase, a simultaneous disto-rotation of the first molars, defined according to Ricketts’ line, was planned together with the application of 2° of additional buccal root torque to both maxillary and mandibular first molars. The same standardized expansion protocol was adopted for both arches, whereas intrusion mechanics, alignment, and torque control of incisors were specifically planned for the lower arch. The planned transverse expansion ranged from 4 to 6 mm and was individualized for each patient based on cusp relationships and the transverse intercuspation between the maxillary and mandibular first molars. Expansion was programmed at a rate of 0.25 mm per aligner stage, without digital overcorrection. Patients were instructed to wear the aligners on a full-time basis, removing them only for meals and oral hygiene procedures, with aligner replacement scheduled every 7 days. Follow-up visits were scheduled at 4-week intervals, during which aligner fit, attachment positioning, and correspondence between the achieved tooth movements and the virtual treatment plan were assessed. The mean number of active aligners used was 60 per arch. In cases requiring additional intraoral scans to optimize appliance fit, new aligners were prescribed to continue and complete the initially approved treatment plan while also preserving the originally planned final tooth positions in the ClinCheck^®^ setup. At the end of treatment, no retention was planned. Dental scans were collected for each patient by using an intraoral scanner iTero^®^ Orthodontic ver. 5.2.1.290 (Align Technology Inc., Santa Clara, CA, USA) before starting treatment (T0), at the end of the treatment (T1), and two-years after the end of treatment with no retention applied (T2). The mean interval between T0 and T1 was 17 ±1.8 months, and between T1 and T2 was 24 ± 1.4 months, which was when the patient had reached permanent dentition.

### 2.5. Measurements

**Figure 1:**
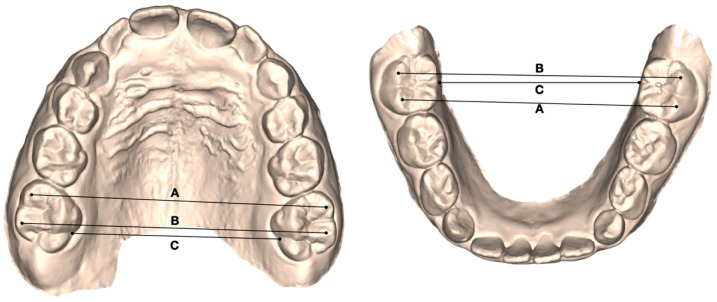
Maxillary and mandibular arch widths measured on digital models at the level of the mesiobuccal cusp tips (**A**) and distobuccal cusp tips (**B**) of the first permanent molars, as well as at the palatal/lingual sulci of the first permanent molars (**C**).

**Figure 2:**
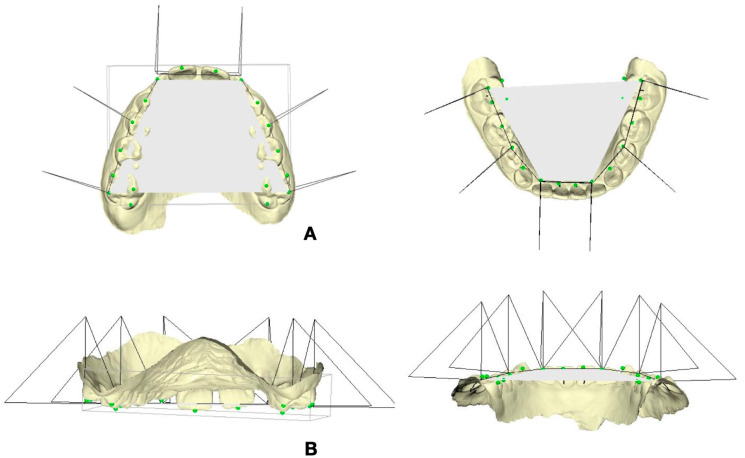
(**A**) Molar torque (UR-6, UL-6, LR-6, LL-6) was assessed as the angle between a triangular reference plane defined by three palatal landmarks (the interincisal papilla and the right and left molar points located at the tooth–gingival margin between the two cusps) and the facial axis of the clinical crown (FACC) of the first permanent molars, identified on the buccal surface. (**B**) Incisor torque (UR-1, UL-1, LR-1, LL-1) was assessed as the angle between the same triangular palatal reference plane and the facial axis of the clinical crown (FACC) of the central incisors.

**Figure 3:**
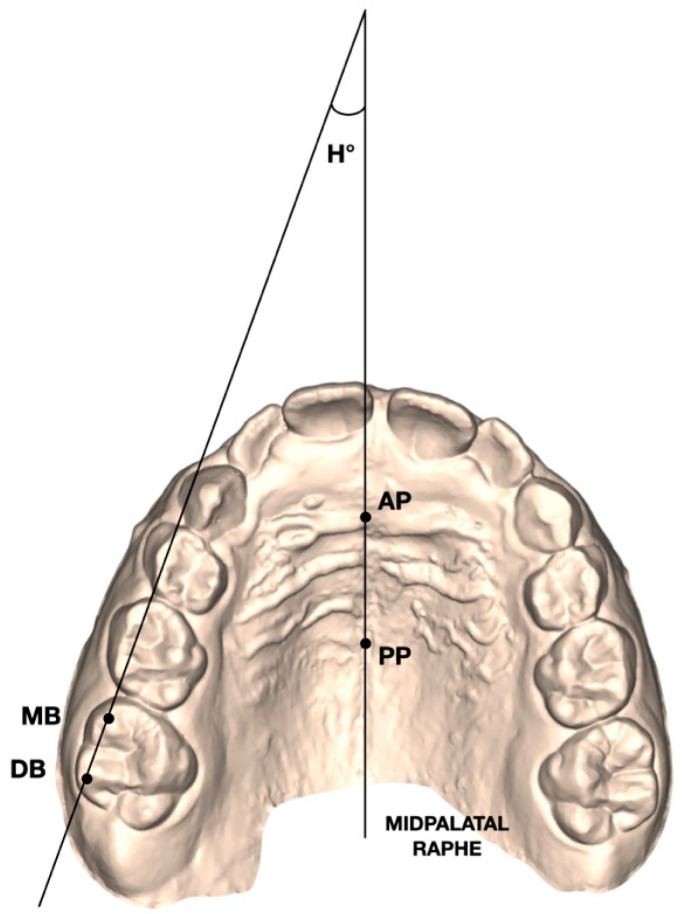
Henry’s angle (H°) measured between the midpalatal raphe (identified by an anterior AP and a posterior PP point along the midpalatal raphe) and the line passing through the mesial buccal (MB) and distal buccal (DB) cusps of the maxillary first molars.

**Figure 4:**
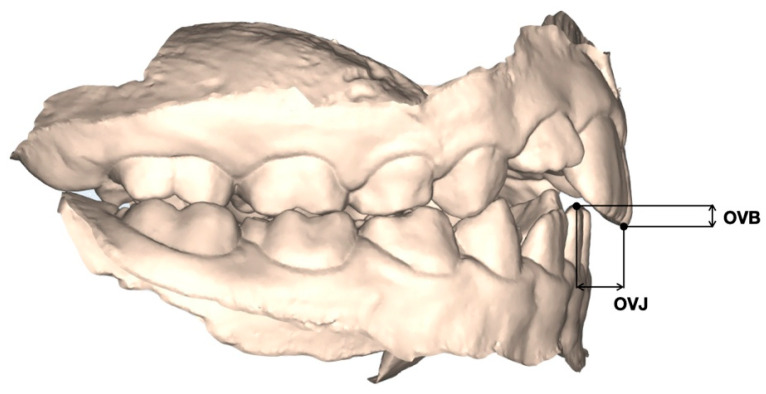
Overbite (OVB), vertical distance between the upper and lower incisors; Overjet (OVJ), horizontal distance between the upper and lower incisors.

Digital dental casts (.stl files) were obtained from the intraoral scans at three observation periods. The reference points were drawn on digital dental casts at T0, T1, and T2 by a single operator (SL), and later checked by a second operator (FG). Angular and linear measurements were collected at all observation time points for both the maxillary and mandibular arches using Viewbox 4.0 software (dHAL Software, Kifissia, Greece) [31,32]. The following transverse parameters were assessed (Figure 1):

- Mesial intermolar width (6–6 mesial): linear distance between the mesiobuccal cusp tips of the permanent first molars;
- Distal intermolar width (6–6 distal): linear distance between the distobuccal cusp tips of the permanent first molars;
- Transpalatal intermolar width (6–6 transpalatal): linear distance between the palatal grooves of the permanent first molars.

- Molar torque (UR-6, UL-6, LR-6, LL-6): angular measurement defined as the angle formed between a triangular reference plane constructed using three palatal landmarks (the interincisal papilla and the right and left molar points located at the tooth–gingival margin interface between the two cusps) and the facial axis of the clinical crown (FACC) of the first permanent molars, identified on the buccal surface (Figure 2A);
- Incisor torque (UR-1, UL-1, LR-1, LL-1): angular measurement defined as the angle between the same triangular palatal reference plane and the facial axis of the clinical crown (FACC) of the central incisors, assessed on the labial surface (Figure 2B).

- Henry’s angle (H°): angular measurement defined as the angle formed between the midpalatal raphe—identified by an anterior (AP) and a posterior (PP) reference point—and the line connecting the mesiobuccal (MB) and distobuccal (DB) cusp tips of the maxillary first molars (Figure 3).

- Overbite (OVB): vertical distance between the upper and lower incisors (Figure 4);
- Overjet (OVJ): horizontal distance between the upper and lower incisors (Figure 4).

- Little index: horizontal distances between the anatomic contact points of the adjacent anterior teeth.

### 2.6. Statistical Analysis

Sample size estimation was performed according to the approach described by Whitehead et al. [33]. Assuming an expected effect size of 1, corresponding to a clinically meaningful change of 2.3° with a standard deviation of 1.2 for the primary outcome (Henry’s angle), a minimum sample of 15 subjects was required to ensure a statistical power of 80% with a two-sided significance level of 5%. Changes in arch measurements across the three observation time points (T0, T1, and T2) were analyzed using Friedman’s analysis of variance for repeated measures [34,35]. When significant differences were detected, pairwise comparisons were carried out using the Wilcoxon signed-rank test [36]. All statistical analyses were performed using R software (version 4.5.1), and the level of statistical significance was set at α = 0.05. Intra-examiner reliability was assessed by randomly selecting twelve dental casts, which were traced and measured twice by the same examiner (SL) with a two-week interval between measurements. Method error was evaluated using Kendall’s coefficient of concordance (W), chosen due to the non-normal distribution of the data, to quantify agreement between repeated measurements.

## 3. Results

### 3.1. T1-T0 Changes

At the end of the T1-T0 interval (Table 1), a statistically significant improvement in transversal width was observed in both upper and lower arches. More specifically, an increase in all the linear transversal variables was found (U6–6 mesial, +2.1 mm; U6–6 distal, +1.8 mm; U6–6 transpalatal, +1.2 mm; L6–6 mesial, +2.4 mm; L6–6 distal, +2 mm; L6–6 transpalatal +1.6 mm). Significant torque changes were also observed at the level of upper first molars (UR-6, +2°; UL-6, +2.1°) and maxillary incisors (U-1, −1.4°). In the lower arch, a significant torque increase in the left first molar was found (LL-6, +2.3°), whereas at the level of the right molar and lower incisors no significant modifications were observed (LR-6, +1.7°; L-1, −0.2°). As for the Henry angle, a significant disto-rotation of the upper first molars was revealed (1.6, −6.8°; 2.6, −5.5°). The short-term observation also highlighted a significant decrease in overjet values (OVJ, −1.5 mm) and a non-significant improvement in the overbite (+0.2 mm). Little’s index significantly decreased at the end of active treatment (*p* < 0.001), reaching values < 0.5 mm.

### 3.2. T2-T1 Changes

In the T2-T1 interval (Table 1), a slight relapse was found in most of the examined variables. In the upper arch, a reduction in the transversal linear measurements was detected without any statistical significance (U6–6 mesial, −0.1 mm; U6-6 distal, −0.3 mm; U6-6 transpalatal, −0.3 mm). Moreover, in the lower arch, none of the transversal variables reported significant modifications (L6-6 mesial, −0.6 mm; L6-6 distal, −0.5 mm; L6-6 transpalatal, −0.5 mm). Regarding the crown angulation values, a significant relapse was reported only at the level of the lower left molar (−1.1°). The other crown angulation values did not show any significant variations in either arch (UR-6, −0.1°; UL-6, −0.5°; U-1, + 0.3°; LR-6, −0.8°; L-1, + 0.3°). The T1-T2 comparison of overjet, overbite and Henry’s angle did not report any statistically significant changes (OVJ −0.4 mm; OVB 0.1 mm; 1.6, −1.6°; 2.6, −1.1°). No significant relapse of Little’s index was detected during follow-up.

### 3.3. T2-T0 Changes (2-Year Follow Up)

The T2-T0 comparison (Table 1) showed statistically significant changes for some of the analyzed variables. More specifically, U6-6 mesial (+2 mm), U6-6 distal (+1.5 mm), L6-6 mesial (+1.6 mm), and L6-6 distal (+1.52 mm) reported a statistically significant increase after 2 years following the end of treatment. U6-6 and L6-6 transpalatal did not reveal any modifications (respectively, +0.9 mm and 1.1 mm). Regarding the crown angulation values, most of the measurements revealed statistically significant changes (UR-6, +1.9°; UL-6 +1.6°; U-1, −1.1; LR-6, +0.9°; LL-6 +1.2°). It was only the lower incisor torque that did not change during the post-treatment follow-up (L-1, +0.1°). The T2-T0 comparison revealed a significant improvement of Henry’s angle on both sides (1.6, −8.4°; 2.6, −6.6°). A significant reduction in the overjet was observed (−1.9 mm), whereas no significant changes in the overbite were reported (+0.3 mm). No significant systematic or random errors were found for any of the detected variables, with values generally low and close to zero. Kendall’s coefficient of concordance test showed almost perfect agreement with a score greater than 0.95 for all linear and angular measures. The T2-T0 comparison confirmed a stable reduction in Little’s index (*p* < 0.001).

## 4. Discussion

The study herein aimed to assess the long-term stability of dental arch changes obtained by means of clear aligner treatment in early mixed dentition. The findings of this investigation demonstrated the efficacy and the stability of clear aligners in producing significant transversal expansion and favorable torque modifications in both upper and lower arches, with a minimal relapse at the 2-year follow-up. In the short term (T1-T0), statistically significant increases in transversal width were observed in all measured parameters. According to our findings, previous studies underscored the efficacy of clear aligners in promoting transversal development during growth [21,22,23,24,25,26,27,37]. Levrini et al. [24] reported a transversal alveolar expansion of +1.16 mm at the level of the upper first permanent molars after about 8 months of treatment, concluding that the expansion obtained can be considered similar to the one achieved using slow maxillary expanders. Moreover, the applied “molars move first” expansion protocol produced a stable and controlled expansion of the posterior segments, with a more pronounced gain in mesial intermolar width (U6–6 mesial, +2.1 mm; L6-6 mesial, +2.4 mm) compared to the distal one (U6–6 distal, +1.8 mm; L6–6 distal, +2 mm). Recently, Lione et al. [21,30] described similar findings by highlighting that this expansion staging involves, during transversal mechanics, the simultaneous movement of the buccal tip and disto-rotation around the palatal root. As a matter of fact, significant disto-rotations of the upper first molars were revealed on both sides (1.6, −6.8°; 2.6, −5.5°). This early activation of the first permanent molars allows the correction of mesio-palatal rotations of the first molars and easier arch development at the level of the lateral segments, which likely eased incisor alignment. Anterior crowding frequently occurs during the early stages of dentition, particularly during the transition from the primary to the mixed dentition, as the eruption of the permanent incisors typically requires at least 1.6 mm of additional space to allow for proper alignment within the dental arch [27]. In this developmental phase, mild to moderate crowding is often observed and can be clinically assessed using Little’s irregularity index (LII) [29], which quantifies the degree of misalignment between the contact points of the mandibular anterior teeth. This index, although originally developed for permanent dentition, can be adapted for use in mixed dentition to provide a reliable estimate of anterior irregularity when only the permanent incisors are present. In cases of mild crowding, interceptive orthodontic approaches such as dental arch expansion may be sufficient to recover the space needed for the correct eruption and alignment of permanent teeth, reducing the likelihood of more complex interventions later on [37,38,39,40,41,42,43,44,45].

In this regard, our findings suggest that the interceptive clear aligner protocol was not only effective in promoting transversal arch development, but also clinically relevant in the management of anterior irregularity. Indeed, Little’s index showed a marked improvement from baseline to the end of active treatment (T0: 7.5 ± 0.4 mm; T1: 0.4 ± 0.1 mm), reaching values indicative of clinically negligible irregularity (LII < 0.5 mm). Moreover, this improvement was maintained at long-term follow-up as LII remained stable during the observation period (T2: 0.4 ± 0.2 mm), supporting the long-term stability of incisor alignment achieved in early mixed dentition.

As for torque modifications, significant results were reported only in the upper arch. More specifically, an increased crown angulation was observed at the level of upper first molars (UR-6, +2°; UL-6, +2.1°), whereas maxillary incisors showed reduced torque values (U-1, −1.4°). On the contrary, Cretella et al. [32] underscored good control of the posterior crown angulation without any statistically significant changes. These findings have been justified by the authors with the planned overcorrection by 2 degrees of extra buccal root torque to overcome the dental tipping. On the contrary, McNamara et al. [16], in a short- and long-term evaluation of RME effects, highlighted an increase in the crown angle of 4.8° at the end of the active phase. It is likely that the greater magnitude of planned expansion in this study contributed to the higher increase in torque values observed. Furthermore, the greater increase in transversal expansion was related with a significant reduction in the incisor crown angulation (U-1, −1.4°). It is well established that in clear aligner therapy, transversal expansion is often associated with a reciprocal retraction of the anterior teeth, leading to a reduction in torque values [36]. Indeed, our findings also revealed a significant overjet reduction of about −1.5 mm at the end of the active treatment. This aspect makes clear aligner treatment particularly favorable in certain clinical conditions, such as Class II malocclusion with increased overjet, for the early reduction of the dental discrepancy. Conversely, in cases where an increase in incisor torque is desired, planning should include a degree of overcorrection to compensate for the predictable loss of torque during treatment [38]. From T1 to T2, all the detected variables remained stable, with only a statistically significant relapse at the level of the lower left molar crown angulation (−1.1°). This suggests that the expansion achieved with clear aligners can be largely maintained over time, even without the use of fixed or removable retention appliances. The lack of significant relapse in Henry’s angle during the follow-up further supports the stability of the molar derotation effect. The T0-T2 comparison confirmed the persistence of most treatment effects after two years, particularly regarding transversal widths and crown angulation. These findings are in line with previous reports on the long-term effects of slow expansion devices [17,18,19], suggesting that clear aligners may represent a valid alternative in managing transversal discrepancies during early mixed dentition. Additionally, the significant reduction in overjet values was stable at follow-up, indicating that sagittal improvements can also be maintained over time. While the literature on clear aligner treatment in growing patients is still limited and predominantly focused on short-term outcomes [21,22,23,24,25,26,27], our results provide encouraging evidence for their long-term efficacy and stability. Compared to traditional rapid or slow expansion appliances, aligners offer a less invasive and more esthetic treatment option, with the added advantage of better hygiene management and potentially greater patient compliance.

Nevertheless, some limitations of this study should be acknowledged. The relatively small sample size and the retrospective design may limit the generalizability of the findings and reduce the power to detect smaller effect sizes. In addition, the absence of a control group treated with conventional appliances does not allow for direct comparisons of treatment effects and stability outcomes. Furthermore, since all patients were treated during the growth phase, physiological craniofacial growth may have partially contributed to the observed changes, and its influence cannot be completely separated from the effects of the orthodontic intervention. Therefore, further prospective studies with larger cohorts, appropriate control groups, and longer follow-up periods are needed to validate these results.

## 5. Conclusions

This investigation supports the effectiveness of clear aligners in achieving stable dental arch modification in early mixed dentition. Despite the lack of retention, most of the achieved dentoalveolar effects remained stable over a 2-year follow-up period, highlighting the potential of clear aligner therapy as a reliable tool in early interceptive orthodontics. However, the retrospective design, small sample size, lack of a control group, and potential influence of growth warrant cautious interpretation. Further prospective controlled studies are needed.

## Figures and Tables

**Table 1:** Descriptive and statistical comparisons of the means (T0-T1, T1-T2, T0-T2) with the Friedman test for repeated measurements followed by the Wilcoxon signed-rank test.

									Comparison of the Means					
Variables	T0		T1		T2		X^2^	*p*	T1-T0		T2-T1		T2-T0	
	Mean	SD	Mean	SD	Mean	SD			Mean	SD	Mean	SD	Mean	SD
U6-6 mesial (mm)	49.7	2.9	51.8	2.8	51.7	2.5	22.8	***	2.1 **	2.3	−0.1	1.9	2 **	1.9
U6-6 distal (mm)	52.0	2.3	53.8	1.8	53.5	1.6	21.5	***	1.8 **	1.6	−0.3	1.4	1.5 **	1.6
U6-6 transpalatal (mm)	34.8	2.2	36	1.8	35.7	2.3	8.4	**	1.2 **	2	−0.3	1.1	0.9	2.2
L6-6 mesial (mm)	44.1	2.7	46.3	2.5	45.7	2	23.4	***	2.4 **	2.6	−0.6	2.4	1.6 **	2.6
L6-6 distal (mm)	46.2	2.7	48.2	2.6	47.7	2	19.6	***	2 ***	1.4	−0.5	2.3	1.5*	1.9
L6-6 transpalatal (mm)	33.0	2.1	34.6	2.1	34.1	1.7	20.2	***	1.6 **	1.4	−0.5	1.8	1.1	1.5
UR-6 torque (°)	−19.3	2.7	−17.3	2.4	−17.4	2.5	28.4	***	2 **	1	−0.1	0.2	1.9 **	0.9
UL-6 torque (°)	−19.3	1.8	−17.2	2.2	−17.7	2.4	26.8	***	2.1 **	2.1	−0.5	2.1	1.6 ***	1.2
U-1 torque (°)	8.6	3.2	7.2	2.9	7.5	3	30.5	***	−1.4 ***	0.7	0.3	0.5	−1.1 ***	0.2
LR-6 torque (°)	−38.1	4.3	−36.4	5.1	−37.2	3.7	14.2	**	1.7	2.3	−0.8	1.9	0.9 *	3.8
LL-6 torque (°)	−38.9	3.4	−36.6	4.1	−37.7	3.7	32.1	***	2.3 ***	2.1	−1.1**	0.8	1.2 ***	1
L-1 torque (°)	−2.5	0.8	−2.7	0.9	−2.4	0.9	9	*	−0.2	0.6	0.3	0.6	0.1	0.7
Henry angle 1.6 (°)	17.7	4.9	10.9	3.8	9.3	3.2	28	***	−6.8 **	3.7	−1.6	3.4	−8.4 **	5.1
Henry angle 2.6 (°)	16.6	3.3	11.1	3.4	10.0	3.8	29	***	−5.5 ***	3.2	−1.1	3.1	−6.6 ***	4.4
Overjet (mm)	5.7	2.9	4.2	2	3.8	1.3	14.3	**	−1.5 **	1.8	−0.4	1.3	−1.9 *	4.4
Overbite (mm)	2.7	1.5	2.9	1.0	3.0	1	1.3	NS	0.2	1.1	0.1	0.7	0.3	2
Little index	7.5	0.4	0.4	0.1	0.4	0.2	-	***	−7.1 ***	0.3	0.0	0.2	−7.1 ***	0.4

SD: standard deviation; X^2^: Friedman chi-squared; *** = *p* < 0.001; ** = *p* < 0.01; * = *p* < 0.05; NS = not significant.

## Data Availability

The datasets used and/or analyzed during the current study are available from the corresponding author on reasonable request.

## Abbreviations

The following abbreviations are used in this manuscript:

ANOVA	Analysis of variance
AP	Anterior point on midpalatal raphe
CA	Clear aligners
DB	Distobuccal
FACC	Facial axis of the clinical crown
IPR	Interproximal reduction
L6–6	Lower intermolar width (first permanent molars)
LII	Little’s irregularity index
LL-1	Lower left central incisor
LL-6	Lower left first permanent molar
LR-1	Lower right central incisor
LR-6	Lower right first permanent molar
MB	Mesiobuccal
OVB	Overbite
OVJ	Overjet
PP	Posterior point on midpalatal raphe
RME	Rapid maxillary expansion
SD	Standard deviation
T0	Baseline
T1	End of treatment
T2	Two-year follow-up
U-1	Upper central incisor
U6–6	Upper intermolar width (first permanent molars)
UL-1	Upper left central incisor
UL-6	Upper left first permanent molar
UR-1	Upper right central incisor
UR-6	Upper right first permanent molar
